# Bioelectrical impedance phase angle and nutritional status in children with intestinal failure on prolonged parenteral nutrition^☆^

**DOI:** 10.1016/j.jped.2023.12.006

**Published:** 2024-04-10

**Authors:** Victória A. Alves, Juliana M. Giesta, Vera L. Bosa, Helena A.S. Goldani

**Affiliations:** a Post-Graduate Program of Child and Adolescent Health, Hospital de Clinicas de Porto Alegre, Universidade Federal do Rio Grande do Sul, Porto Alegre, RS, Brazil; b Department of Nutrition, Hospital de Clinicas de Porto Alegre, Universidade Federal do Rio Grande do Sul, Porto Alegre, RS, Brazil; c Post-Graduate Program of Child and Adolescent Health, Faculdade de Medicina, Hospital de Clínicas de Porto Alegre, Universidade Federal do Rio Grande do Sul, Porto Alegre, RS, Brazil

**Keywords:** Phase angle, Bioelectrical impedance, Child, Intestinal failure, Short bowel syndrome, Parenteral nutrition

## Abstract

**Objective:**

To compare the phase angle (PhA) through bioelectrical impedance (BIA) of children with intestinal failure (IF) using prolonged parenteral nutrition (PN) followed by an Intestinal Rehabilitation Program, with a control group.

**Methods:**

Children under 10 years of age with IF using prolonged PN for >60 days (study group) were included. The control group consisted of healthy children without chronic pathologies, matched by sex and age. Anthropometric parameters evaluated were: weight, height, weight/age z-score (W/A), height/age z-score (H/A), BMI, BMI/A z-score, arm circumference, triceps skinfold, subscapular skinfold, mid-arm muscle circumference. BIA parameters were resistance (R), reactance (Xc), and phase angle (PhA).

**Results:**

Twenty-eight children were included in the study group, median (IQR) age was 11 (8–27) months, 53.6 % were male. In the control group, 28 children were included, median (IQR) age was 12.5 (8–24.7) months, 50 % were male. Children from the study group had W/A z-scores and H/A z-scores significantly lower than controls. There was no significant difference between PhA in the study group and controls, [median (IQR) 4.3° (3.8;4.6) vs 4.0° (3.8;5.4) respectively, *p* = 0.980]. Prematurity was significantly higher in the study group than in the controls, but there was no significant correlation between gestational age at birth and PhA of the children from the study group.

**Conclusion:**

Children with IF using prolonged PN showed lower W/A and H/A compared to the control group, but without significant difference between the PhA of children with IF compared to controls.

## Introduction

Intestinal failure (IF) is characterized by functional reduction of the intestine and severe malabsorption of nutrients, resulting from surgical resection of the intestine, intestinal congenital anomalies, and functional or intestinal motility disorders.^1^ The most frequent cause of IF is short bowel syndrome (SBS), defined as the need for prolonged parenteral nutrition (PN) for a minimum period of two months after extensive intestinal resection.^2^ The most frequent causes of SBS are necrotizing enterocolitis, intestinal atresia, gastroschisis, volvulus and long-segment Hirschsprung disease (2). The prognosis of SBS is influenced by many factors, including the length, anatomy and function of the remaining bowel.^1^ The incidence of SBS has been estimated at 24.5/100,000 births per year, but it can reach 7/1000 births in premature newborns with a birth weight of <1500 g.^1^

In recent decades, there has been an advance in the survival of patients with IF using prolonged PN, reaching over 90 % in European and North American centers.^2^, ^3^, ^4^ In southern Brazil, a five-year survival rate of 90.4 % was recently found.^5^ The advent of Multidisciplinary Intestinal Rehabilitation Programs is one of the main factors responsible for the good results in the short and long term.^6^^,^^7^

Children with chronic IF who require prolonged PN are at risk for altered body composition and impaired muscle function and strength, possibly due to malnutrition, malabsorption, and reduced physical activity due to frequent hospitalization.^8^ They are also at risk for low bone mineral density, leading to abnormal physical development and reduced linear growth.^9^

Bioelectrical impedance (BIA) is a technique capable of clinically estimating the body composition, mainly to assess muscle mass and hydration,^10^ and with application in pediatric patients.^11^ The method consists of the passage through the body of an electric current of low amplitude and high frequency providing data on phase angle (PhA), resistance (R), reactance (Xc).^10^

PhA is the most clinically used as its prognostic value has been assessed in terms of nutritional assessment, overall mortality, cause-specific mortality, medical and surgical complications, length of hospital stay, and use of healthcare resources.^10^ Several studies have evaluated PhA in children with various conditions such as liver disease, inflammatory bowel disease, congenital heart disease, genetic disorders, renal disease, rheumatic disease, and critically ill children.^11^, ^12^, ^13^, ^14^, ^15^, ^16^

PhA is an indicator of the individual's health, as well as the integrity of the cell membrane and body cell mass.^17^ A low PhA value suggests cell death or decreased integrity of cell membranes,^17^ while high values are compatible with a large number of intact membranes and good cell function.^14^ PhA has shown a great ability to predict outcomes in several conditions, and it varies depending on age and sex, and is related to lean body mass. PhA increases progressively over the first two decades of life and is higher in male adolescents than females, particularly after the age of 13 years. Less consistent evidence has been reported in younger subjects.^18^

Regarding the patients with IF, it has been shown that the PhA predicted the number of hospital readmissions, length of hospital stays and mortality in adult patients with SBS on long-term PN.^19^ However, to date, no study has assessed the PhA in children with IF using prolonged PN. Therefore, the aim of this study was to evaluate the PhA through BIA of children under 10 years old with IF using prolonged PN, taking as reference a control group of healthy children without chronic pathologies, matched by sex and age.

## Methods

### Study population

This is a cross-sectional study, which evaluated the PhA through BIA of children under 10 years of age diagnosed with IF using prolonged PN, followed by the Intestinal Rehabilitation Program for Children and Adolescents at a tertiary public university hospital in Southern Brazil.

The control group consisted of children who underwent minor surgical procedures such as correction of inguinal hernia, hydrocele and tympanostomy enrolled at the Otorhinolaryngology and Pediatric Surgery outpatient clinic of the same hospital; and healthy children without chronic pathologies who were under the care of a municipal Family Health Strategy unit and a municipal School for Early Childhood Education. The use of a control group aimed at comparing some anthropometric parameters and data resulting from BIA, due to the lack of standard curves for healthy children in this age group.

The inclusion criteria were: a) study group - children aged under 10 years old with a diagnosis of IF, currently using prolonged PN for a period equal to or greater than 60 days, stable and without acute infections; b) control group - children without chronic diseases, matched by age and sex, aged between zero and 10 years.

The exclusion criteria were: a) study group - children unaccompanied by a person responsible for family recognition, children with genetic syndromes or multiple comorbidities, including renal impairment or decompensated liver disease; b) control group - children with gastrointestinal, hepatorenal, endocrine, neurological, cardiac diseases, severe congenital malformations, genetic syndromes, pneumopathies, and any diseases that predispose to chronic nutritional impairment.

### Data collection

The collection period was from June 2019 to November 2022. The children's demographic data and clinical information were collected from the medical records and/or through an interview with the parents or legal guardians.

The anthropometric parameters collected were weight and height according to the Brazilian Society of Pediatrics (SBP).^20^ Body mass index (BMI) was calculated based on weight and height. BMI for age (BMI/A), weight for age (W/A), and height for age (H/A) were evaluated using the World Health Organization curves and Anthro calculator.^21^ The nutritional diagnosis was established from the W/A and BMI/A curve in “underweight”, “eutrophic” and “overweight” for z-score “< −2”, “≥ −2 and < +2” and “≥ +2”, respectively; for diagnosis based on the H/A curve, “short stature” and “eutrophic” were established for z-scores “< −2” and “≥ −2”.

To measure the arm circumference (AC), an inextensible and inelastic measuring tape was used in each region measured three times, using the average of the values. The triceps skinfold (TST) and subscapular skinfold (SST) were performed in each region three times, and after that, the mean values were calculated, using a scientific Cescorf® adipometer calibrated at a constant pressure of 10 g/mm².^20^ The mid-arm muscle circumference (MAMC) was calculated as AC–(3.14 x TST), indicating the muscle tissue reserve, without correcting the bone area.^21^

All anthropometric/skinfold measurements were performed by two operators (VAA, JMG), equally well-trained nutrition therapists, and with the same equipment to achieve the best possible accuracy.

### Bioelectrical impedance (BIA)

The BIA test was carried out with a four-pole *Biodynamics device*, model 450, Seattle, WA, USA,^16^^,^^22^ operating through the passage of alternating current of low frequency and high voltage (800 mA and 50 kHz), painless and completely safe.

The examination was carried out according to the protocols indicated by the manufacturer. All children were in a supine position for at least 10 min before and during the BIA measurements.^23^ Adhesive-backed electrodes were placed on the dorsal side of the hand and foot on the same side of the body. All BIA measurements were performed by the same operator (VAA), who had special training for the use of the equipment. All children had a 4-hour fast, and the children from the study group had their PN cycled off for at least 4 h to avoid the influence of the electrolyte content of the administered fluid.^24^ All children were in clinically stable conditions, normally hydrated, without edema, ascites, or daily weight gain not exceeding recommended levels, ensuring a stabilized fluid status.

### Variables of the study

The study variables analyzed were: age; sex; gestational age at birth; prematurity considered as gestational age at birth <37 weeks; days of PN use; causes of IF; SBS classification; length of remaining bowel (RI); and presence of ileocecal valve (ICV). The PN dependence ratio (PNDI) from the children on PN was defined as the ratio of non-protein energy intake (NPEI) to resting energy expenditure (REE). This PNDI ranges from mild PN dependence (< 80 %) to high PN dependence (> 120 %).^25^

The anthropometric parameters evaluated were weight, W/A z-score, height, H/A z-score, BMI, BMI/A z-score, AC, TST, SST, and MAMC. The BIA parameters evaluated were resistance (R), reactance (Xc), and phase angle (PhA). Phase angle was calculated by using the following formula: phase angle = (Xc/R) × (180/π).^14^

According to the anatomical classification of SBS, patients were classified as having SBS type I – terminal jejunostomy; type II – remnant intestine with jejuno-colon anastomosis without ileocecal valve (ICV); type III – remnant intestine with jejuno-ileo-colon anastomosis and preservation of ICV.^2^

The results of the control group and study group were matched according to sex and age. For all children with a history of prematurity, chronological age was corrected according to gestational age at birth up to two years of age.^26^

### Statistical analysis

Quantitative variables were described as mean and standard deviation (±SD) or median and interquartile range (IQR). The Shapiro-Wilk test was applied to assess the normality of the variables. For variables that did not show significance in this test, the mean (±SD) was used, and the *t*-Test was applied. For variables that showed significance, indicating a lack of normal distribution, the median (IQR) was used, and the Mann-Whitney U test was applied. Categorical variables were described by absolute and relative frequencies. When comparing proportions, Student's *t*-test, Pearson's chi-square test, or Fisher's exact test were used. The significance level used was *p* < 0.05 and the analyses were performed using the Statistical Package for Social Sciences (SPSS) version 27.0.

### Ethical aspects

The research was approved by the local Research Ethics Committee, no. GPPG 19-0394, CAAE no. 17877619.8.0000.5327. To participate in the research, the written and informed consent form was signed by the parents or legal guardians of the children in the study group and in the control group.

## Results

A total of 56 children participated in the study, 28 from the study group and 28 from the control group. The characteristics of the study group are presented in Table 1. It was found that the majority of children with SBS had type II (remaining intestine with jejuno-colon anastomosis and without ICV), and 50 % of the children had moderate PN dependence. The most frequent causes of IF were intestinal atresia and volvulus, corresponding to 25 % each.

**Table 1:** Clinical characteristics of children with IF on prolonged PN.

Characteristics	Study group (*n* = 28)
Days of use of PN^1^	451.5 (280; 889.7)
RI^2^ (cm)	37.5 (19.2; 58.7)
SBS classification^3^ (*n* = 26)	
type I	5 (19 %)
type II	14 (53 %)
type III	7 (26 %)
Absence of ICV^4^	19 (73 %)
Causes of IF^5^	
volvo	7 (25 %)
atresia	7 (25 %)
enterocolitis	6 (21 %)
gastroschisis	4 (14.3 %)
long segment Hirschsprung	3 (10.7 %)
other causes	1 (3.6 %)
PN dependence ratio	0.97 (0.8; 1.2)
mild	7 (25 %)
moderate	14 (50 %)
high	7 (25 %)
Enteral nutrition^6^	72.8 % (43.0; 114.8)

Values expressed in median (IQR); frequency n (%).

1 PN – parenteral nutrition.

2 RI – remaining intestine.

3 SBS – short bowel syndrome.

4 ICV – ileocecal valve.

5 IF – intestinal failure.

6 percentage of enteral feed calories in relation to Recommended Dietary Allowance (RDA) guidelines for energy intake.

The characteristics of the study and control groups are presented in Table 2. The history of prematurity was more frequent in the study group compared to the control group (64.3% vs 17.9 %, *p* = 0.001). Anthropometry and BIA results of the study group and control group are also described (Table 2).

**Table 2:** Anthropometry and BIA parameters of children with IF on prolonged PN and controls.

Variable	Study Group *n* = 28	Control Group *n* = 28	p
Age (months)	11 (8; 27.2)	12.5 (8; 24.7)	0.908
Sex Masc	15 (53.6 %)	15 (53.6 %)	1.000
Prematurity	18 (64.3 %)	5 (17.9 %)	0.001*
Gestational age at birth (weeks)	34.9 ± 4.4	38.2 ± 3.0	0.002*
Weight (kg)	9.2 (7; 12.4)	9.6 (8; 13.2)	0.210
Height (cm)	71.7 (67.1; 88.7)	73.2 (68.5; 89.9)	0.362
W/A^1^ (z-score)	−0.63 ± 1.1	0.3 ± 1.0	0.002*
W/A Rating:			
Underweight	2 (7.1 %)	1 (3.6 %)	
Eutrophic	26 (92.8 %)	27 (96.4 %)	
Overweight	0 (0 %)	0 (0 %)	
H/A^2^ (z-score)	−1.2 ± 1.3	−0.43 ± 0.1	0.023*
H/A Rating:			
Short	6 (21.4 %)	1 (3.6 %)	−0.101
Eutrophic	22 (78.6 %)	27 (96.4 %)	
BMI^3^ (kg/m²)	16.6 ± 1.5	17.4 ± 1.7	0.063
BMI/I^4^ (z-score)	0.05 ± 1.1	0.6 ± 1	0.066
BMI/A Rating:			
Underweight	2 (7.1 %)	0 (0 %)	
Eutrophic	24 (85.7 %)	27 (96.4 %)	0.285
Overweight	2 (7.1 %)	1 (3.6 %)	
AC^5^ (cm)	15.2 ± 2	15.5 ± 2.3	0.530
TST^6^ (mm)	9.4 (7.2; 10)	7 (5.1; 10)	0.104
SST^7^ (mm)	7 ± 3	5.9 ± 1.6	0.077
MAMC^8^(cm)	12.3 ± 2.1	13.1 ± 2.9	0.234
Bioimpedance (BIA)			
Resistance (ohms)	675.9 ± 87.4	713.3 ± 81.1	0.102
Reactance (ohms)	51.0 (44.7; 58.7)	51.7 (47.4; 63.3)	0.283
PhA^9^ (°)	4.3 (3.8; 4.6)	4.0 (3.8; 5.4)	0.980

Values expressed in mean (±SD), median (IQR), frequency n (%).

1 weight for age.

2 height for age.

3 body mass index.

4 body mass index for age.

5 arm circumference.

6 triceps skinfold.

7 subscapular skinfold.

8 mid-arm muscle circumference.

9 phase angle.

⁎ statistically significant. P values were determined using the *t*-Test or Mann-Whitney U test or Fisher chi-square test.

Children from the study group had W/A z-score and H/A z-score significantly lower than the control group. The W/A z-score were within −2 and +2 in 92.8 % of children from the study group and in 96.4 % of controls. The H/A z-score were within −2 and +2 in 78.6 % of children from the study group and in 96.3 % of controls. BMI, BMI/A, AC, TST, SST, and MAMC values did not differ between groups. The body composition results of fat mass and fat-free mass will be presented in another study.

Overall, the PhA was not significantly different between children in the study group and controls. The children were categorized into three age groups: < 1 year, 1 to 3 years, and > 3 years. In children under 1 year old, 15 from the study group and 13 from the control group had a mean (± SD) PhA of 4.5 ± 1.4° and 4.0 ± 0.8°, respectively (*p* = 0.13). For children aged 1 to 3 years, 8 from the study group and 11 from the control group had a mean (± SD) PhA of 4.1 ± 0.4° and 5.1 ± 1.6°, respectively (*p* = 0.14). In children over 3 years old, 4 from the study group and 3 from the control group had a mean (± SD) PhA of 4.8 ± 0.5° and 5.8 ± 0.5°, respectively (*p* = 0.40). No significant differences in PhA were observed between children in the study group and those in the control group across the different age groups. Stratification for ages 8 to 10 years was not performed due to only one patient per group.

Prematurity was significantly higher in the study group than controls (Table 2). Gestational age at birth was significantly lower in children from the study group than controls. However, there was no significant correlation between gestational age at birth and PhA of the children from the study group and controls, *r* = 0.023, *p* = 0.907 and *r* = 0.247, *p* = 0.205, respectively.

## Discussion

This study evaluated the PhA through BIA of children with IF using prolonged PN. There was no significant difference between the PhA of children with IF using prolonged PN in relation to a control group. Additionally, children with IF had shorter stature compared to children from the control group. This is the first study carried out on children from an Intestinal Rehabilitation Program at a public hospital in Brazil.

In terms of prognostic factors of SBS in this population, the RI length <40 cm and the absence of ICV have been associated with long-term dependence on PN.^27^ In the present study, the majority (53 %) of children with SBS had type II, 26 % type III, and 19 % type I; these data showed the distribution of anatomical types of SBS, all with a prognosis of need for long-term PN use. All children from the study group were receiving PN, with a median PN dependence ratio of 0.97. Among them, 25 % had mild, 50 % had moderate, and 25 % had high dependence on PN. In clinical practice, the PN dependence ratio reflects intestinal sufficiency more accurately than enteral feeding which may be impaired by malabsorption.^28^ Additionally, all of them were also receiving enteral feeds orally, via nasogastric tube, or through gastrostomy. The types of diet varied, including amino acid-based formulas, with extensively hydrolyzed proteins, polymeric formulas, and solid foods. The median calorie intake of enteral feeds was 72.8 % of the recommended dietary allowance (RDA) guidelines for energy intake.

Regarding the anthropometric parameters evaluated, almost all children were eutrophic, with W/A z-scores within −2 and +2 in 92.8 % of the study group and 96.4 % of controls. The H/A z-scores were within −2 and +2 in 78.6 % of the study group and 96.3 % of the controls. Children in the study group had significantly lower W/A and H/A z-scores compared to the control group. BMI, BMI/A, AC, MAMC, TST, and SST values did not show significant differences between children with IF and controls.

Regarding the reference values for PhA in children, some studies have described reference curves for PhA in the age group from four years of age onwards.^18^^,^^29^^,^^30^ The PhA values found in the present study did not differ significantly between children with IF and controls, even when categorized into three age groups: < 1 year, 1 to 3 years, and > 3 years. Stratification for ages 8 to 10 years was not performed due to the low number of patients. Both children with IF and controls had an average above 4.0°, which might indicate a favorable prognosis for patients with IF and adequate cellular integrity.^14^

Another study carried out with children with a median age of 4.8 years from an intensive care unit, demonstrated that those with PhA below 2.8° had lower survival compared to those above 2.8°.^31^ In the population of adults with IF, Fassini et al*.*^30^ found no significant difference between the PhA of patients with IF and controls, mean of 5.7° vs 6.4°, respectively. Similarly, the present study found no significant difference between children with IF and controls. These data may indicate that patients with IF, when evaluated in a stable clinical condition with satisfactory nutritional status, might experience a positive clinical evolution.

Prematurity is one of the risk factors for intestinal failure-associated liver disease.^32^ Although prematurity was significantly higher in the study group than controls, there was no significant correlation between gestational age at birth and PhA of the children from the study group.

In the present study, anthropometry and PhA assessed through BIA showed that the nutritional therapy used in children with a mean age of 11 months and a mean duration time of PN use of 1 year and 2 months, seemed to be adequate when compared to the group control. Although patients may have been dehydrated and malnourished in the past, their current status may reflect a nutritionally compensated and balanced state, reinforcing the importance of care by a multidisciplinary team well evidenced in the literature.^6^^,^^7^

A limitation of the study may be related to the reduced number of patients; however, IF is a rare condition with low prevalence, and multicentric studies should be performed. Another limitation of the study might be related to the accuracy of BIA data acquisition, particularly concerning the hydration status, as it may influence bioimpedance measurements. The authors tried to minimize this effect as much as possible by following the recommendations of a 4-hour fast.^23^ PN infusion was cycled off at least 4 h before the BIA acquisition to avoid the influence of intravenous administration of fluids, which occurs mainly in the 2 h following the suspension of fluid administration.^24^ Nonetheless, to our knowledge, this is the first study that evaluated PhA through BIA in Brazilian children with IF using prolonged PN.

The findings of this study showed that children with IF using prolonged PN followed by an Intestinal Rehabilitation Program had satisfactory nutritional conditions in relation to healthy children. Further studies are needed to evaluate the PhA in a long-term follow-up fashion since the initiation of parenteral therapy, as many children may present with malnutrition during this period. BIA is a non-invasive and bedside procedure, which could be easily included in the clinical workup and performed on a routine basis for children with IF. PhA in addition to other parameters of BIA might be useful to obtain information on body composition and cellular health during the follow-up of those patients to identify patients at nutritional risk.

## Conflicts of interest

The authors declare no conflict of interest.
